# Interactions of the protease inhibitor, ritonavir, with common anesthesia drugs

**DOI:** 10.1111/pan.14529

**Published:** 2022-07-24

**Authors:** Anders Svedmyr, Henrik Hack, Brian J. Anderson

**Affiliations:** ^1^ Dept Anaesthesia Starship Children's Hospital Auckland New Zealand

**Keywords:** anesthesia, antiviral, children, COVID‐19, drug interactions, pharmacodynamics

## Abstract

The protease inhibitor, ritonavir, is a strong inhibitor of CYP 3A. The drug is used for management of the human immunovirus and is currently part of an oral antiviral drug combination (nirmatrelvir–ritonavir) for the early treatment of SARS‐2 COVID‐19‐positive patients aged 12 years and over who have recognized comorbidities. The CYP 3A enzyme system is responsible for clearance of numerous drugs used in anesthesia (e.g., alfentanil, fentanyl, methadone, rocuronium, bupivacaine, midazolam, ketamine). Ritonavir will have an impact on drug clearances that are dependent on ritonavir concentration, anesthesia drug intrinsic hepatic clearance, metabolic pathways, concentration‐response relationship, and route of administration. Drugs with a steep concentration‐response relationship (ketamine, midazolam, rocuronium) are mostly affected because small changes in concentration have major changes in effect response. An increase in midazolam concentration is observed after oral administration because CYP 3A in the gastrointestinal wall is inhibited, causing a large increase in relative bioavailability. Fentanyl infusion may be associated with a modest increase in plasma concentration and effect, but the large between subject variability of pharmacokinetic and pharmacodynamic concentration changes suggests it will have little impact on an individual patient, especially when used with adverse effect monitoring. It has been proposed that drugs that have no or only a small metabolic pathway involving the CYP 3A enzyme be used during anesthesia, for example, propofol, atracurium, remifentanil, and the volatile agents. That anesthesia approach denies children of drugs with considerable value. It is better that the inhibitory changes in clearance of these drugs are understood so that rational drug choices can be made to tailor drug use to the individual patient. Altered drug dose, anticipation of duration of effect, timing of administration, use of reversal agents and perioperative monitoring would better behoove children undergoing anesthesia.

## 1 INTRODUCTION

Ritonavir has been used for decades for human immunovirus (HIV) infection in adults and children. It inhibits the HIV proteases that are necessary to cleave long protein chain precursors into mature smaller infectious HIV proteins. The consequent cytochrome P‐450 (CYP) 3A inhibition and its implication for anesthesia providers are described.^1^, ^2^, ^3^ Interest in this drug has resurfaced because antiviral agents (e.g., remdesivir, molnupiravir, and nirmatrelvir) have gained prominence for community‐based early management of proven COVID‐19 infection.^4^ Nirmatrelvir is combined with the protease inhibitor, ritonavir, that slows the metabolism of nirmatrelvir via CYP enzyme inhibition. When used as combination therapy with ritonavir, nirmatrelvir half‐life is doubled.^5^ The inclusion of ritonavir in any drug combination is likely to create complexities and restrictions for its use.^6^

Ritonavir is a strong inhibitor of CYP 3A, an enzyme system that comprises the majority of adult human liver cytochrome P‐450 and metabolizes a broad range of drugs, including those commonly used in anesthesia. Resources are available (https://www.covid19‐druginteractions.org/) that detail ritonavir interactions where raised substrate drug concentrations are associated with serious and/or life‐threatening reactions or when potential for loss of virologic response and possible resistance may occur. These interactions are often based on in vitro information, are poorly quantified for many drugs used in anesthesia and were reported with higher doses associated with ritonavir use for human immunodeficiency virus treatment when compared with those currently used for viral COVID‐19 management. Table 1 demonstrates drugs relevant to pediatric anesthesia with current anesthesia practice recommendations.

**TABLE 1:** Ritonavir effect on commonly used anesthetic drugs. Ritonavir is a potent inhibitor of CYP 3A and a weaker inhibitor of CYP 2D6 mediated metabolism. Ritonavir is an inducer of CYP 1A2, CYP 2B6, glucuronosyl transferase (UGT), and possibly CYP 2C9 and CYP 2C19. There are no known interactions of ritonavir with those plasma esterases responsible for metabolizing remifentanil.

Fentanyl	Plasma clearance is decreased. Adverse effects monitoring advocated
Alfentanil	Plasma clearance is decreased. Respiratory monitoring advocated
Remifentanil	No known interaction
Morphine	No interaction of clinical import
Methadone	Plasma clearance reported to be slightly decreased Oral bioavailability increased
Oxycodone	Clearance reduced. Oral bioavailability increased Reduce oral dose, monitor effects
Rocuronium	Neuromuscular blockade prolonged. Neuromuscular monitoring required. Consider sugammadex for reversal of neuromuscular effect.
Atracurium	No interaction
Propofol	No interaction
Ketamine	Prolongation of clinical effect anticipated. Effect monitoring advocated
Midazolam	Oral bioavailability increased Clearance reduced. Consider dose adjustment if repeat dosing or infusion, monitor effects
Bupivacaine	Plasma clearance is decreased but limited clinical relevance after single dose
Lidocaine	Plasma clearance is decreased but limited clinical relevance after single dose. Caution if running an infusion
Clonidine	No interaction
Dexmedetomidine	Unlikely reduced clearance, monitor effect as usual practice
Ondansetron	No interaction
Acetaminophen	No interaction
Ibuprofen	No interaction

A full list of drug interactions with ritonavir is available from the UK NIH COVID‐19 treatment guidelines (Paxlovid HCP fact Sheet 03182022.pdf, https://www.covid19treatmentguidelines.nih.gov/). A comprehensive guide to drugs that can be compromised during ritonavir administration has been published by Manzolini and colleagues.^7^ These drugs can be grouped using a traffic light system that ranges from red (do not co‐administer) through orange (potential interaction) to yellow (weak interaction) and green (no interaction expected). Drugs discussed in this current review fall under the “red or orange” classification.^7^

Current guidelines from pediatric anesthesia societies often recommend postponement of nonessential anesthesia and surgery in children until at least 14 days after onset of COVID‐19 symptoms.^8^, ^9^ This delay avoids anesthesia in the early course of the disease when nirmatrelvir–ritonavir might be given. There will be occasions when the need for medical intervention cannot be postponed and anesthesiology practitioners should be mindful of potential interactions between ritonavir and drugs used during anesthesia and adapt perioperative care appropriately.

We review drugs interactions with ritonavir in order to help guide anesthetic practice. Pharmacokinetic–pharmacodynamic simulation is used to demonstrate impact of interactions and why some interactions may have little clinical importance while others have major clinical impact. We demonstrate that most, if not all, anticipated effects of drug interactions relevant to anesthesia could be anticipated and dealt with if the nature those interactions were better understood.

## 2 RITONAVIR PHARMACOKINETICS

Ritonavir is a potent synthetic protease inhibitor. The drug has good oral absorption with high bioavailability. Ritonavir is primarily metabolized by cytochrome (CYP 3A) isozymes and, to a lesser extent, by CYP 2D6. The apparent oral clearance (CL/F) in adults averages 7–9 L h^−1^ with a volume of distribution (Vd/F) of 20–40 L. Ritonavir has high protein binding (98%–99%) to both albumin and α1‐acid glycoprotein. Doses that have been used for the management of HIV (ritonavir 600 mg b.d., C_MAX_ 11.2 mg L^−1^, C_MIN_ 3.7 mg L^−1^) in adults are greater than those currently used for COVID‐19 (100 mg b.d., C_MAX_ 0.89 mg L^−1^, C_MIN_ 0.22 mg L^−1^). Plasma concentrations reach steady‐state 2 weeks after starting treatment, this delay may possibly be attributed to auto induction of enzyme pathways.^10^

Children (median age, 7.7 years; range, 0.5–14.4 years) have 30% higher per kilogram ritonavir clearance estimates compared with adults.^11^ The increased apparent per kilogram clearance can be explained using allometric theory.^12^ Dose‐related nausea, diarrhea, and abdominal pain were the most common toxicities in infants and children (6 weeks PNA—12 years) treated for HIV.^11^, ^13^

## 3 DRUG INTERACTIONS

The enzyme inhibition reported with ritonavir is instantaneous but the magnitude of effect is related to plasma concentration and the shape of the concentration‐response curve.^14^, ^15^, ^16^ Other drugs that use the cytochrome clearance pathways may also be affected by ritonavir. The drug is a potent inhibitor of CYP 3A but is minimally affected by other CYP 3A inhibitors (e.g., ketoconazole), reflecting ritonavir high CYP 3A4 receptor affinity. It also inhibits CYP 2D6–mediated metabolism, but to a lesser extent.

Ritonavir is also an inducer of several enzymes (CYP 1A2, CYP 2B6, glucuronosyl transferase [UGT], and possibly CYP 2C9 and CYP 2C19), although this induction has slower onset. Enzyme induction through activation of DNA transcription requires 7–14 days to achieve a maximum effect. The magnitude of these interactions remains poorly quantified.

### 3.1 Contributing factors affecting CYP inhibition

Ritonavir irreversibly inhibits CYP 3A4 enzyme activity leading directly to a reduction in clearance that is concentration dependant.^14^ As a consequence, inhibition takes several days to reverse as it requires de novo enzyme synthesis to restore baseline metabolic activity. It is classified as mechanism‐based irreversible inhibitor.^15^ The reported in vitro concentration of the drug that inhibits activity by 50% (IC_50_) ranged up to 1.62 mg L^−1^ for CYP3A.^17^, ^18^ The IC_50_ for CYP 2D6 was 1.8 mg L^−1^ while that for the CYP 2C family ranged from 5.8 to 21.6 mg L^−1^.^17^

The largest interactions occur with drugs that are extensively metabolized by CYP 3A where there is high intrinsic hepatic clearance. Ritonavir competitive effects are less for drugs with intermediate clearances (10 to 80 L h^−1^/70 kg) and any effect is minimal for drugs with a low intrinsic clearance (<10 L h^−1^/70 kg).

The inhibitory concentration will only have impact on clearance of the drug if it is cleared predominantly by that pathway. Methadone, for example, is cleared by cytochrome P450 mixed oxidases (CYP3A4, CYP2B6, CYP2C9) and clearance was minimally affected by ritonavir. It is probable that any decrease in CYP3A4 activity has minimal effect or is ameliorated by clearance from other pathways.^10^

While clearance is an important parameter that describes drug elimination, termination of effect from anesthesia induction drugs (propofol, etomidate, thiopentone) is due to redistribution and not clearance. Many drugs are administered as intravenous infusion in anesthesia practice and redistribution has major impact on observed plasma concentration after ceasing infusion.^19^ A decrement time such as the context‐sensitive half‐time (CSHT) after a 1‐h infusion of fentanyl is approximately 20 min, which increases to 270 min after an 8‐h infusion in adults.^20^ Fentanyl clearance was reduced by 67% in adults treated with ritonavir for HIV,^21^ but it is fentanyl concentration that determines analgesic effect and redistribution after infusion may lessen the impact of this ritonavir interaction.

The CYP 3A4 enzyme is present in both the liver and the gastrointestinal wall and inhibition of metabolism in the gut wall will lead to some drugs having greater relative bioavailability when given orally.

### 3.2 The concentration‐response relationship

The shape of the concentration‐response relationship will also have effect on the drug effect observed when clearance is decreased. This concentration‐response relationship is often described using the sigmoid Emax model^22^:

$$\text{Effect}=E_{0}+\frac{E_{\max}\cdot {\mathrm{Ce}}^{N}}{{\mathrm{Ce}}^{N}+C_{50}^{N}}.$$

**FIGURE 1:**
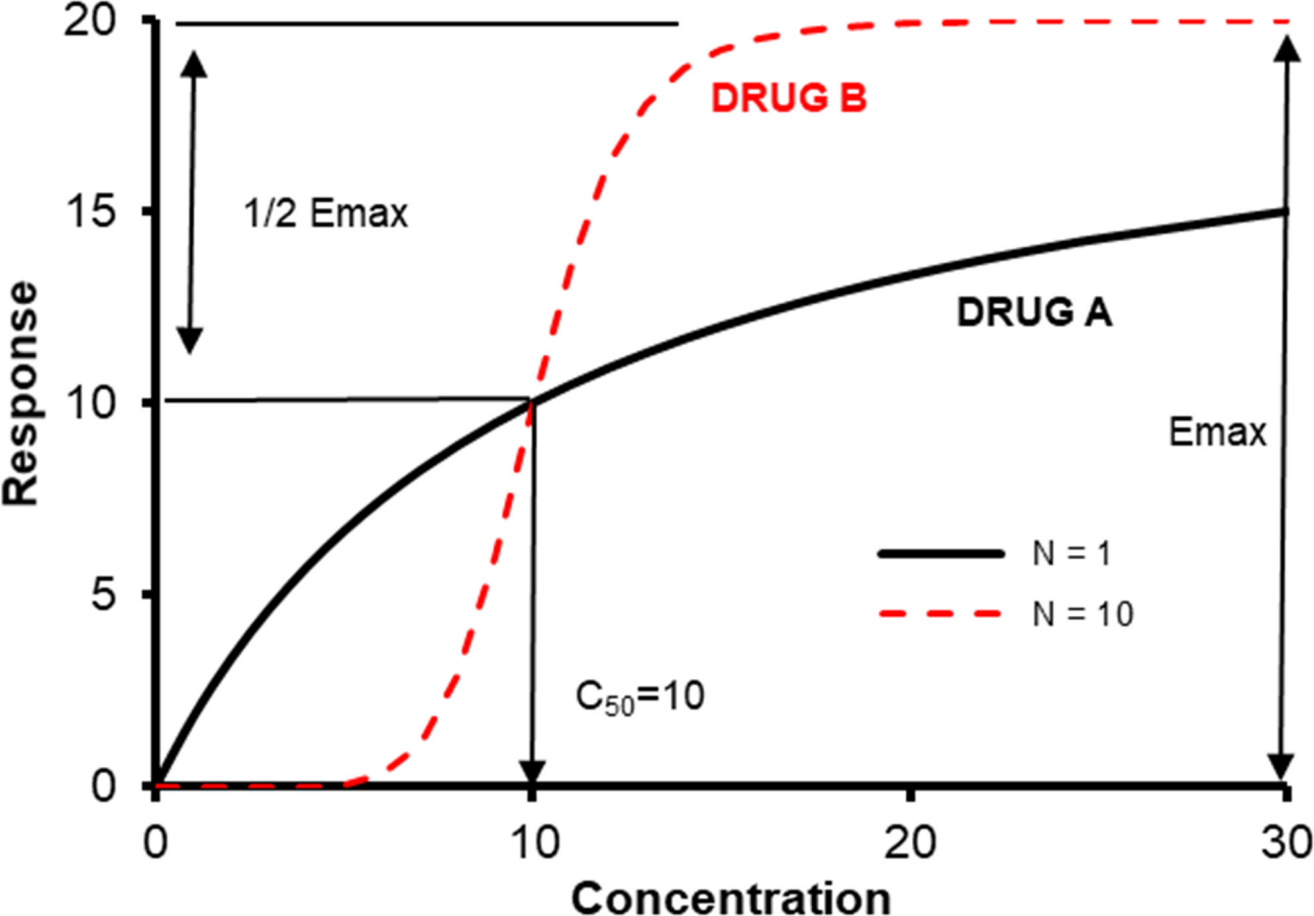
The sigmoid Emax curve showing response curves. Both drugs have the same maximum response (*E*_max_), but the slope of Drug B is steeper and described by a Hill coefficient (*N*) of 10. Small changes in concentration between the C_20_ and C_80_ have pronounced effect. Small changes in concentration when the concentration is above the C_95_ or if the slope is gentle (Drug A) have far less effect.

The C_50_ is the concentration at which the effect is half maximal (*E*_max_), Ce is concentration in the effect compartment. The *E*_0_ parameter accounts for baseline response and N, the Hill coefficient, defines the steepness of the curve slope (Figure 1). Small changes in concentration will have high impact on drugs where the response curve is steep between the C_20_ and C_80_. Contrarily, if a drug is given at a dose 2–3 times its ED_95_ (e.g., rocuronium), then any decrease in concentration will have no effect because the concentration sits on the maximum flat upper part of the sigmoid response curve.^23^

Similar relationships can be plotted for a drug's ability to inhibit the response of another. The IC_50_ is the concentration of an inhibitor where the response (or binding) is reduced by half. Ritonavir administration produced a dose‐dependent CYP 3A inhibition assessed using midazolam *oral* exposure. An exposure–inhibition effect curve for ritonavir was established with an in vivo IC_50_ of 0.43 μg L^−1^.^14^

### 3.3 Offset of competitive effect

Ritonavir is a type II ligand that perfectly fits into the CYP3A4 active receptor site cavity, leaving little leeway for other drugs to get access and bind to the receptor. Maximal inhibition of hepatic and gut CYP 3A4 activity was reported when ritonavir was orally administered in doses of more than 100 mg in adults.^24^ Duration of effect of the drug–drug inhibition will be determined by ritonavir clearance and de novo regeneration of enzyme synthesis. The clinically relevant elimination half‐life (t_1/2_β) is 3–5 h.^10^ Ritonavir 300 mg po b.d. for 9 days achieved a target concentration of 10 mg L^−1^ within 48 h and this concentration was observed to reduce the metabolic clearance of midazolam 30%–50%; the effect persisted after ceasing ritonavir and was 27% baseline at 72 h.^25^ With a competitive inhibitor, it might be reasonable to allow 4–5 half‐lives to elapse for drug elimination. However, although ritonavir could then be assumed cleared by 24 h after completing the 5‐day course of nirmatrelvir/ritonavir; duration of effect is longer because enzyme synthesis is required to restore baseline enzyme activity. Strong inducers of CYP3 A4 (e.g., carbamazepine) can compromise ritonavir activity by increasing synthesis of the enzyme.^7^

## 4 RITONAVIR IMPACT ON COMMON ANESTHESIA DRUGS

### 4.1 Experience with HIV antiviral therapy and anesthesia

Ritonavir and its effect on clearance of some anesthetic drugs through CYP 3A is well recognized.^1^, ^2^, ^3^ Drugs that are not metabolized by the CYP 3A enzyme system (propofol, atracurium, remifentanil, inhalational agents) have been suggested as preferred options for anesthesia. It is suggested that anesthetic drugs affected by CYP 3A inhibition should be titrated carefully.^1^ There are concerns about antiarrhythmic drugs (amiodarone, quinidine, disopyramide, calcium channel blockers) that might cause major cardiovascular toxicity when co‐administered with ritonavir and that these drugs should be used with extreme caution. Titration and extreme caution are not so easily achievable unless the drug is short acting and administered by infusion.

Most anesthesia drugs are given as an intravenous bolus and dose is determined by volume of distribution rather than the clearance that describes elimination. Clinical scenarios where inhibition of CYP 3A affected anesthesia are rarely reported. Reports exploring the frequency of perioperative adverse events are limited. No increased risk for critical respiratory events for patients undergoing treatment with protease inhibitors were observed in 1900 HIV‐positive patients undergoing surgery.^26^ This was unexpected because the plasma clearance of opioids such as fentanyl and neuromuscular blocking drugs (NMBDs, for example, rocuronium) are known to be prolonged.^21^

### 4.2 Probable drug interactions

The inhibition of CYP 3A enzyme by ritonavir may impair the metabolism of frequently used anesthetic and analgesic drugs such as midazolam, fentanyl, ketamine, rocuronium, and bupivacaine.^3^ These drugs have intermediate intrinsic clearance (midazolam 21 L h^−1^/70 kg,^27^ fentanyl 30 L h^−1^/70 kg,^28^ ketamine 60 L h^−1^/70 kg,^29^ rocuronium 17.8 L h^−1^/70 kg,^30^ bupivacaine 35 L h^−1^/70 kg^31^). Magnitude and clinical impact of these clearance changes remain uncertain.

Bupivacaine is metabolized by both CYP 1A2 and CYP 3A4. Ketamine^32^ rocuronium^30^ and midazolam^33^ have all have steep concentration‐response curves (Hill coefficient >3) and small concentration changes due to clearance change can have large effect consequences. Midazolam is commonly given orally as a premedicant and is metabolized in the gastrointestinal tract wall as it crosses the intestinal mucosa, decreasing bioavailability.^34^

Oxycodone metabolism is through CYP3A‐mediated N‐demethylation to noroxycodone and CYP2D6 O‐demethylation to oxymorphone and noroxymorphone. Clearance is in the intermediate range (48 L h^−1^/70 kg)^35^ and because it is metabolized by both CYP 3A and CYP 2D6, clearance reduction by ritonavir will occur. However, this opioid, like the benzodiazepine, midazolam, has increased relative bioavailability when given orally with ritonavir and a smaller oxycodone dose will be needed to avoid opioid‐related adverse effects.^36^

Ritonavir reduced the clearance of fentanyl in adults by 67% when given in doses similar to those used for HIV infection.^21^ Fentanyl is commonly given as either infusion or intermittent bolus dose, situations where redistribution rather than clearance may have greater effect on plasma concentration. While it is possible that ritonavir concentrations achieved in children using the lower dose that augments nirmatrelvir activity are less than the IC_50_ 1.62 mg L^−1^ associated with CYP 3A4 inhibition,^17^, ^18^ fentanyl clearance will still be compromised with the implication that fentanyl plasma concentration will be slow to decrease after an intravenous bolus and that accumulation may occur during infusion.

Protease inhibitors also can inhibit specific uridine 5′‐diphosphoglucuronosyltransferase (UGT) pathways. This accounts for the increase in bilirubin concentration (i.e., UGT 1A1 responsible for bilirubin conjugation) observed in some patients, although UGT 1A6 (i.e., acetaminophen glucuronidation) and UGT 2B7 (i.e., morphine glucuronidation) are unaffected. Dexmedetomidine is metabolized in the liver by UGT 1A4 and UGT 2B10 and it is unlikely to have reduced clearance; dexmedetomidine has additional clearance through aliphatic hydroxylation (CYP 2A6), and N–methylation CYP 2D6.^37^

Protease inhibitors are substrates and inhibitors of drug transporters such as P‐glycoprotein transporters. The coagulation status with the concomitant use of warfarin, which is metabolized by CYP 2C9, may be altered by enzyme induction (e.g., ritonavir).^38^ Protease inhibition of P‐glycoprotein transporters has impact on coagulation after dabigatran use.^39^ Coagulation should be monitored when using these oral anticoagulants.

### 4.3 Pharmacokinetic–pharmacodynamic simulation of inhibition

Simulation was used to explore the impact of ritonavir on ketamine, rocuronium, fentanyl, and midazolam as exemplars to demonstrate the influence of ritonavir on effect or cautions advocated in Table 1. An assumption of a 30% clearance reduction attributable to ritonavir was made because observed concentrations after the 100 mg b.d. po dose were lower that the reported IC_50_ estimates.^17^, ^18^ Simulations were performed in Berkeley Madonna (Robert Macey and George Oster of the University of California, Berkeley, USA) for a typical child 9 years (30 kg) to exemplify PKPD time related changes. Allometry was used to scale from adult to child PK parameter estimates.

#### 4.3.1 Ketamine

**FIGURE 2:**
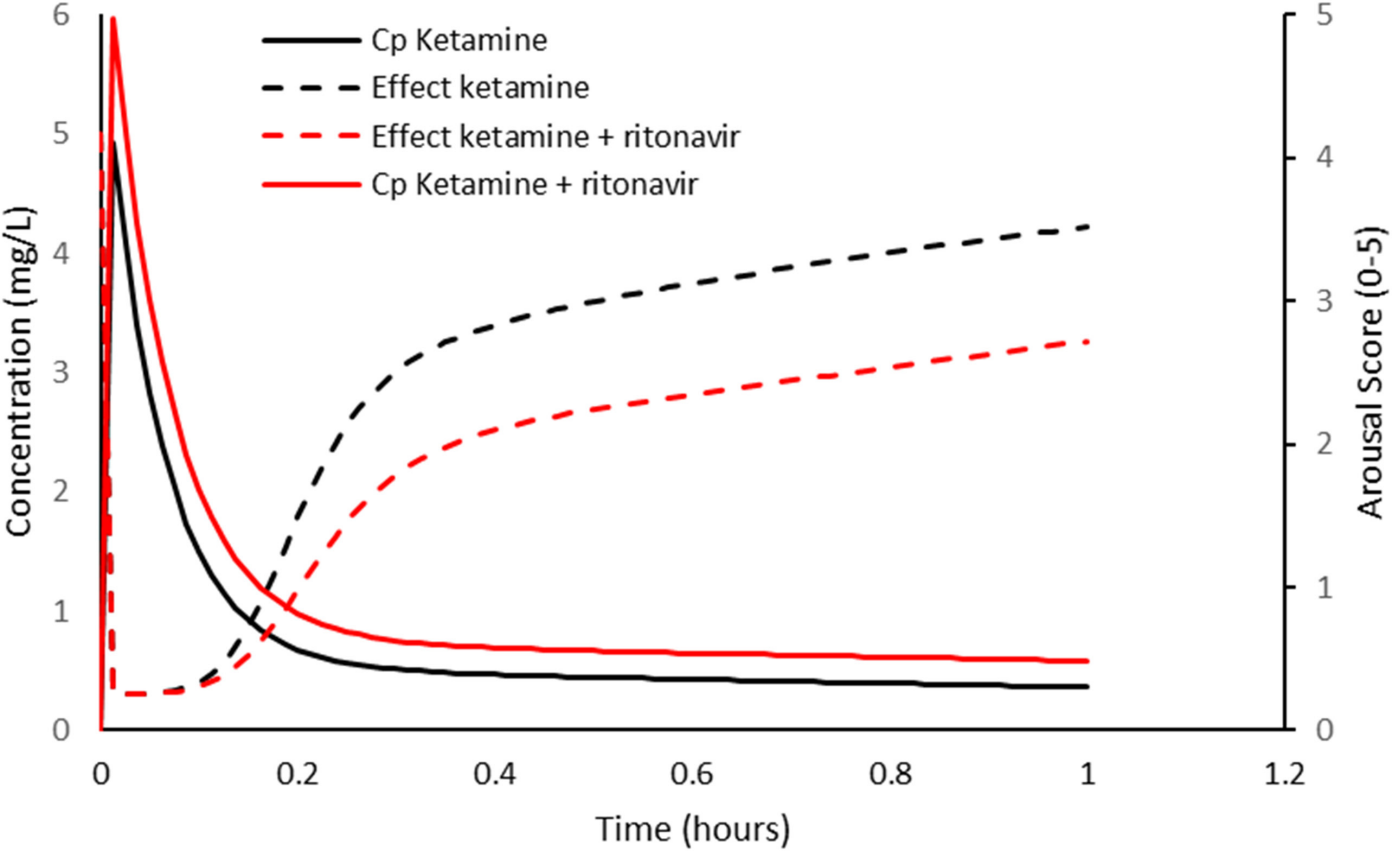
Simulation of plasma concentration (*C*_p_) and sedation score in a 9‐year‐old, 30‐kg child given intravenous ketamine 1.5 mg kg^−1^ when clearance is doubled. The arousal score is graded from 0 to 5 where a score of 2 indicates the child arouses slowly to consciousness, with sustained painful stimulus and a score of 3 indicates the child arouses with moderate tactile or loud verbal stimulus. Pharmacokinetic and pharmacodynamic parameter estimates were from Herd et al.^32^

Ketamine undergoes *N*‐demethylation to norketamine. It is metabolized by CYP 3A4, although CYP 2C9 and CYP 2B6 also have a role. Figure 2 shows a simulation in a child given intravenous ketamine 1.5 mg kg^−1^. The arousal score is graded from 0 to 5 where a score of 2 indicates the child arouses slowly to consciousness, with sustained painful stimulus and a score of 3 indicates the child arouses with moderate tactile or loud verbal stimulus.^32^ It can be seen that the time to reach a score of 2–3 is increased by approximately 30 min when clearance is decreased by 30%. The steepness of the response curve means that small changes in concentration has a notable change in sedation. The converse of this trend (i.e., shortening of time to arousal) has been reported after barbiturate enzyme induction,^40^ because these small concentration changes have major sedation effect consequences.^41^

#### 4.3.2 Rocuronium

**FIGURE 3:**
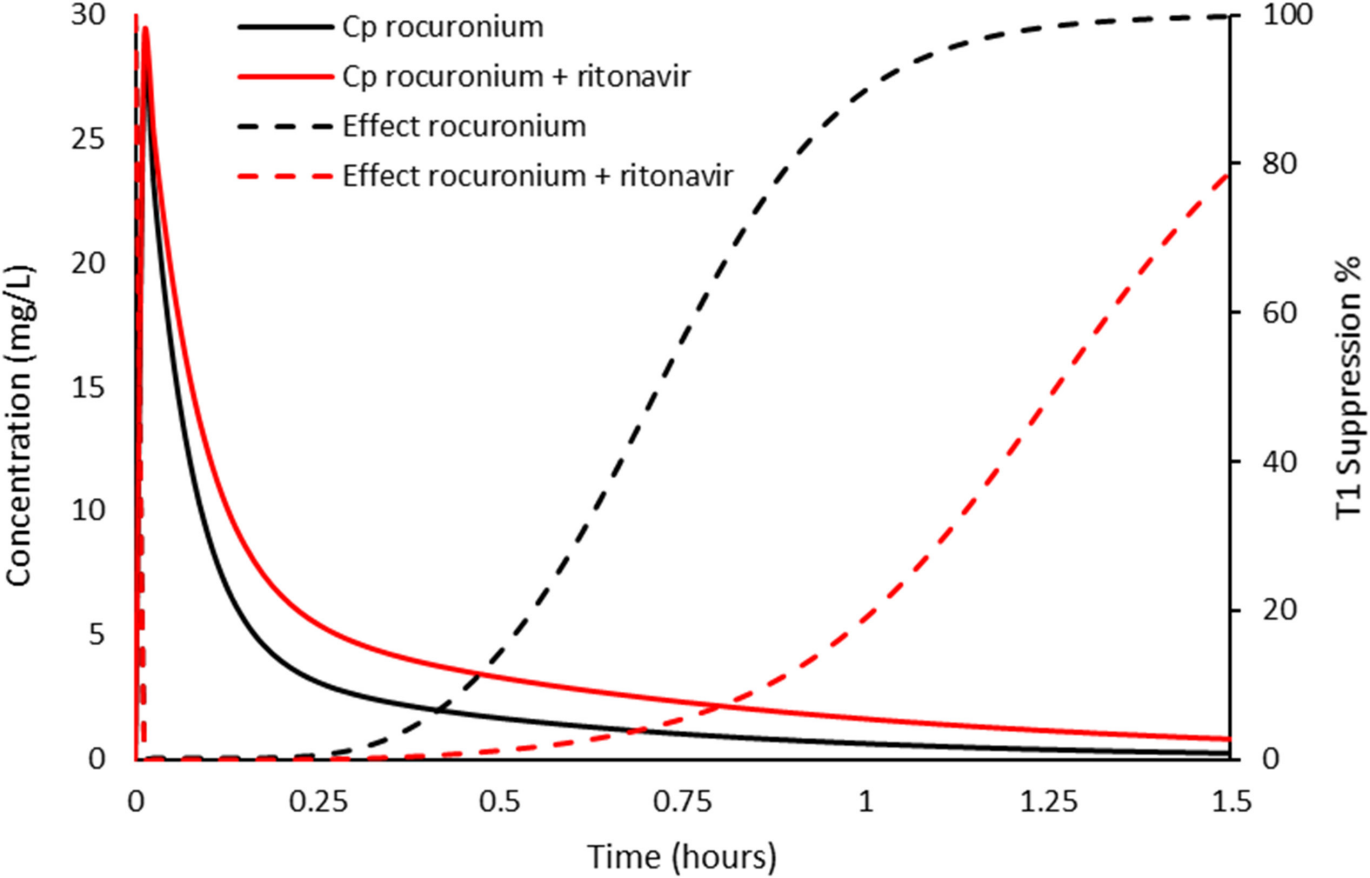
Simulated plasma concentration (*C*_p_) and effect profiles of rocuronium 1.2 mg kg^−1^ (4 × ED_95_) with and without ritonavir. The first twitch of the train‐of‐four response has been used to characterize the time‐effect profile. Recovery is slower when rocuronium is given in the presence of ritonavir and the duration of dense blockade is also longer. Concentrations achieved neuromuscular blockade on the lower flattened upper part of the sigmoid response curve, representing maximum effect (inverted Emax response). PKPD parameter estimates from Vega et al.^30^

The time course of neuromuscular blockade during general anesthesia is commonly monitored using the adductor pollicis muscle. The first twitch of the train‐of‐four response has been used to characterize the time‐effect profile using an integrated population pharmacokinetic–pharmacodynamic model.^30^ That model had a Hill coefficient of 3.9, similar to that reported for ketamine (Hill 3.7).^32^ Figure 3 demonstrates a simulation for a child given a large intravenous dose (4 × ED_95_) to enable rapid intubation. While recovery is slower when rocuronium is given in the presence of ritonavir, the duration of blockade (T1 suppression >90%) is also longer. Concentrations achieved neuromuscular blockade on the flattened upper part of the sigmoid response curve, representing maximum effect. Any concentration decrease after dose would be slower than without ritonavir inhibition and duration of dense blockade prolonged.

#### 4.3.3 Fentanyl

**FIGURE 4:**
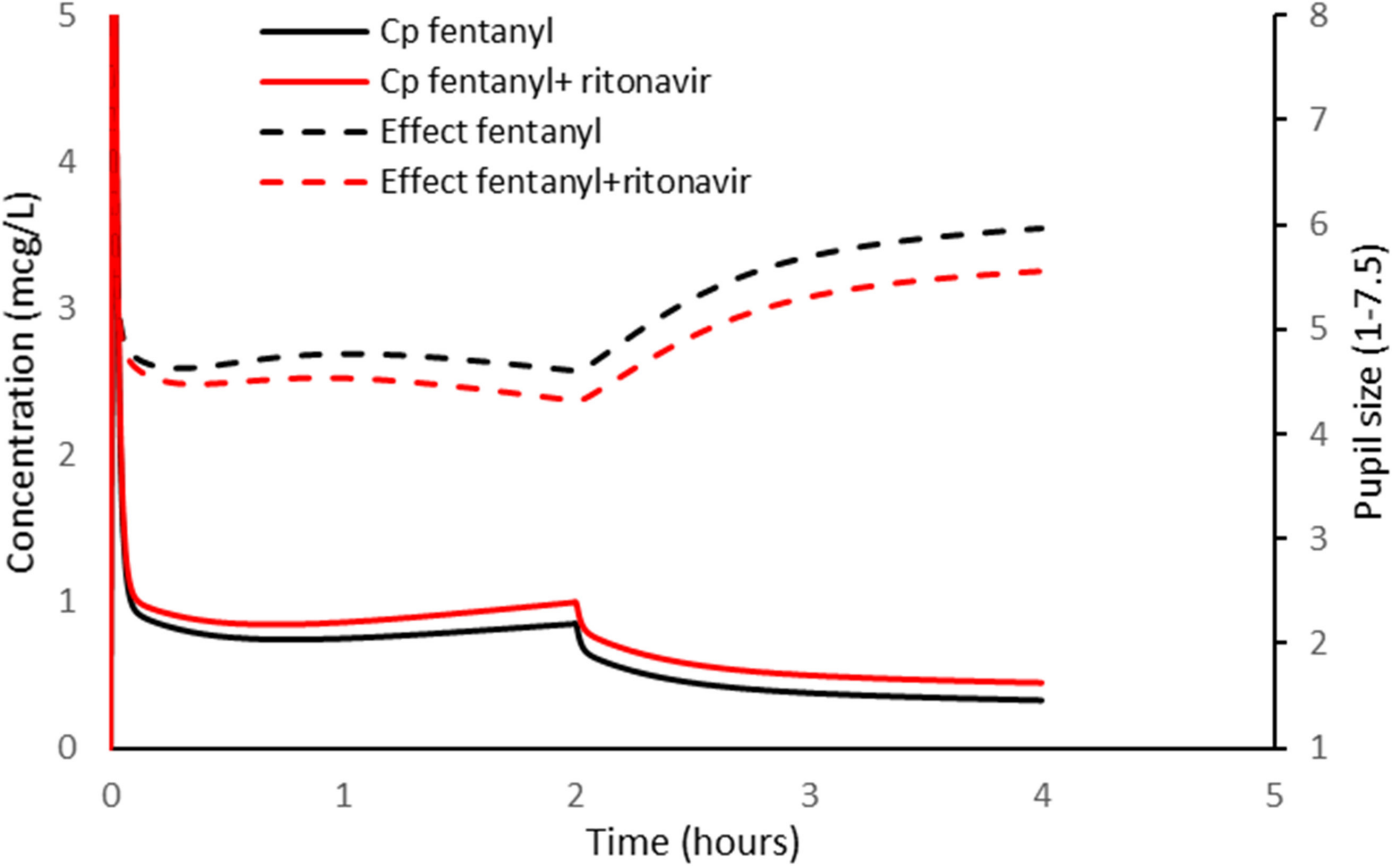
Fentanyl plasma concentration (*C*_p_) is related to pupillary constriction with and without co‐administration of retinovar. Intravenous fentanyl 1 μg kg^−1^ was given as a loading dose. Maintenance was 1 μg kg^−1^ h^−1^ for 2 h. Simulation revealed that small changes in concentration after infusion resulted in small changes in pupillary effect. PK parameter estimates from Shafer et al.^28^ PD parameter estimates from Asbury et al.^42^

Fentanyl clearance is reported to be slower in the presence of ritonavir.^21^ There is no concentration‐analgesic response relationship described for fentanyl in children; the relationship between concentration and pupillary constriction was used as a surrogate (Figure 4).^42^ Simulation revealed that small changes in concentration only resulted in small changes in pupillary effect, attributable to the smaller Hill coefficient (Hill = 1) and redistribution after infusion slowing decline of plasma concentrations. While pupil size and pain have only a loose correlation,^43^, ^44^ pharmacodynamic parameters for both are associated with considerable variability. This variability suggests that these small population differences observed for onset of analgesia or peak effect will have little impact on an individual patient,^45^ particularly because opioids are commonly titrated to effect in an individual and adverse effect monitoring (e.g., respiratory depression) used. Caution should be advocated when fentanyl is used for long duration where drug accumulation could occur if co‐administered with ritonavir.

#### 4.3.4 Midazolam

**FIGURE 5:**
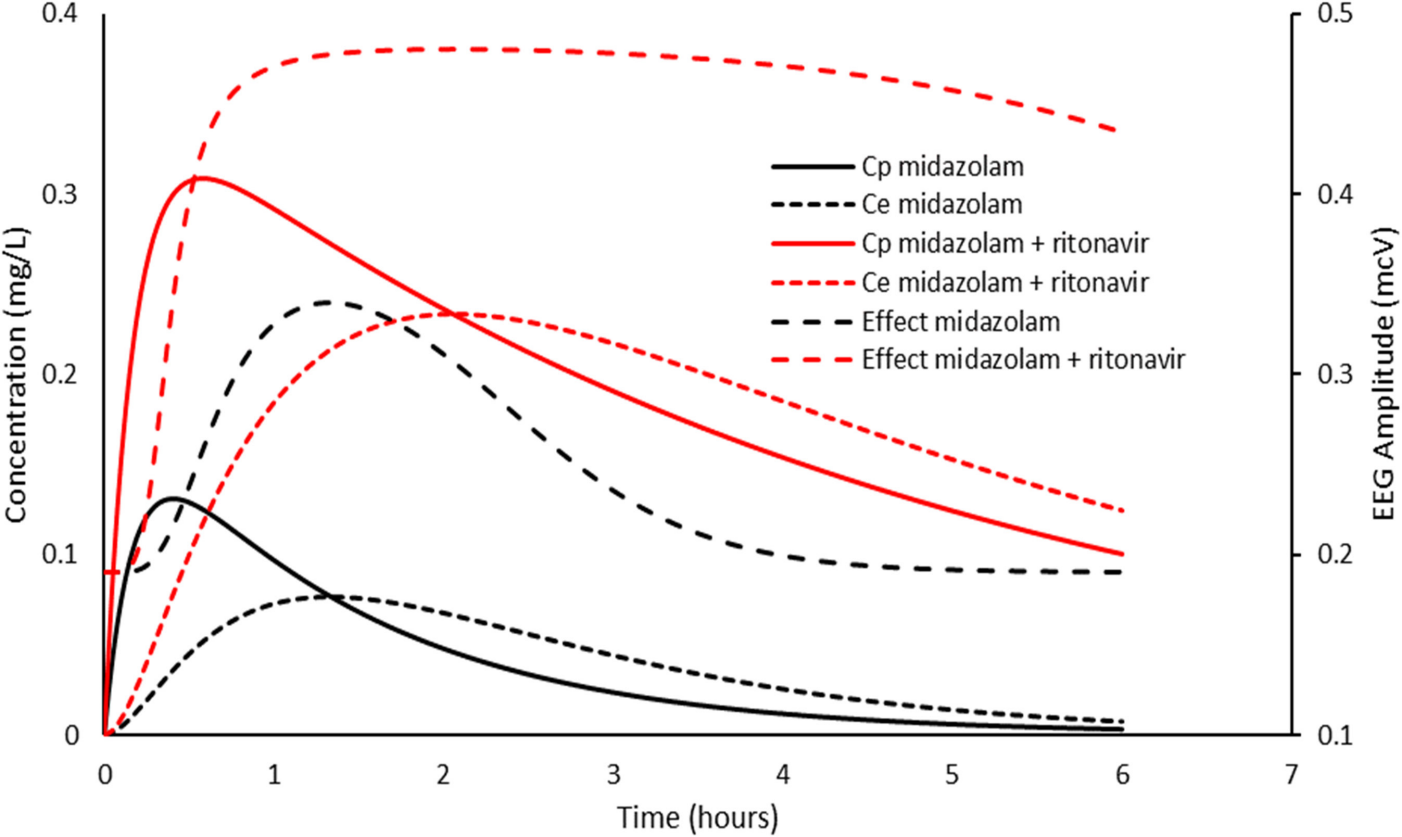
A child was given midazolam 0.5 mg kg^−1^ orally. The effect compartment concentration (*C*_e_) is linked to plasma concentration by a rate constant (keo). Simulation shows the impact of increased bioavailability (twofold) and slower clearance (30% reduction). Pharmacodynamics were described using electroencephalographic amplitudes in the 11.5–30 Hz (beta) frequency band; these were used as a proxy for sedation. Not only is electroencephalographic effect prolonged due to slower clearance, but a ceiling effect is also achieved because of higher concentrations. Pharmacodynamic parameter estimates from Mandema et al.^33^

Midazolam is metabolized mainly by hepatic hydroxylation (CYP 3A4).^46^ Inhibition of this enzyme in the gut increases bioavailability, while inhibition in the liver both lessens the first pass effect and slows clearance.^47^ PKPD relationships have been described for IV midazolam in adults. When an EEG signal is used as an effect measure, the E0 0.19 mcV, *E*_max_ 0.3 mcV, EC_50_ is 77 μg mL^−1^, N 3.1 with a T_1/2_keo of 1 minute.^33^ Figure 5 shows the impact of increased bioavailability (assumed 2‐fold) after oral administration. Not only is electroencephalic effect prolonged due to slower clearance, but a ceiling effect is also achieved because of higher concentrations. Sedation recovery lags behind the decline in plasma concentration more because of the shape of the concentration‐response relationship than due to a lag attributable to effect compartment kinetics.

### 4.4 Transplant recipients

Immunosuppressant drugs such as ciclosporin, tacrolimus, and the mTOR inhibitors (sirolimus, everolimus) are cleared through the CYP 3A enzyme pathway. The use of ritonavir in those receiving treatment for HIV increases concentrations of these drugs rapidly within 1–2 days. High concentrations of tacrolimus can speedily cause adverse effects including kidney injury, seizures, posterior reversible encephalopathy, and death. The implications of a lower dose of ritonavir for COVID‐19 than used for HIV remain speculative. Therapeutic monitoring with subsequent dose reduction to maintain target concentration is imperative.^48^

## 5 OTHER IMPLICATIONS FOR CHILDREN

Children who present for anesthesia are often taking medications for concomitant pathology. The CYP 3A enzyme system is ubiquitous. Rifampicin, a potent CYP 3A inducer, decreased clearance of ritonavir by 35%.^10^ Carbamazepine is another strong inducer of CYP 3A enzyme activity. The implications of using ritonavir short‐term with a child taking long‐term carbamazepine, or other drugs that induce the CYP 3A (dexamethasone, phenobarbitone, phenytoin, rifampicin) are speculative. It could lead to both a risk of toxicity from an increased concentration of the anticonvulsant as well as a potential loss of virological response because of a competitive interaction. Phenytoin dose may require an increase in the presence of ritonavir, possibly attributable to CYP 2C9 inhibition.

Antibiotic (rifambutin, clarithromycin) and antifungal (ketoconazole) concentrations are increased in the presence of ritonavir, but ritonavir itself may be minimally affected by other CYP 3A inhibitors (e.g., efavirenz, fluconazole, ketoconazole, clarithromycin, fluoxetine) because of its high CYP 3A4 receptor affinity relative to these other drugs.

Drugs that are metabolized by CYP 1A2 (e.g., theophylline) or UGT (zidovudine, sulfamethoxazole) have minimal clinical effect from co‐administration of ritonavir because are they weak inhibitors of CYP3A4 (minimal impact on ritonavir) and alternative clearance pathways are available for these drugs so reduction in drug exposure is not large. Use of some antiarrhythmics (flecainide, verapamil, quinidine, nifedipine) should be extremely cautious. Amiodarone, a drug that is known to be metabolized by CYP 3A has large volumes of distribution and low clearance, suggesting minimal impact from a 5‐day course of ritonavir for COVID‐19 on acute use of amiodarone.

## 6 CONCLUSIONS

Interactions with drugs that are considered to be affected by ritonavir during anesthesia may not be as important as assumed once the nature of the interaction is understood and provision made to manage the interaction. Ritonavir dose and consequent plasma concentration influence interaction effect. Observations from when used for HIV infection may have less importance if dose is lower, resulting in a ritonavir concentration that is below the IC_50_ for CYP 3A inhibition. Some drugs (e.g., methadone, aminophylline, bupivacaine) have alternative clearance pathways that can be used if CYP 3A is unavailable. Drugs with a steep concentration‐response relationship (ketamine, midazolam, rocuronium) are mostly affected because small changes in concentration have major changes in effect. An increase in midazolam and oxycodone concentration is observed after oral administration because CYP3A in the gut is inhibited, causing a large increase in relative bioavailability. Fentanyl infusion may be associated with a modest increase in plasma concentration, but the slope parameter for the response relationship is small and the large between subject variability of PK and PD parameters suggests concentration changes will have little impact on an individual patient.^45^ Adverse effect monitoring, as with any strong opioid, remains mandatory.

It has been proposed that anesthesia be avoided during symptomatic COVID‐19 infection or that if anesthesia is necessary then drugs that have no or only a small metabolic pathway involving the CYP 3A enzyme be used in conjunction with nirmatrelvir–ritonavir (e.g., propofol, atracurium, remifentanil, and the volatile agents). Use of nirmatrelvir–ritonavir for the early treatment of SARS‐2 COVID‐19‐positive patients is restrictive; aged 12 years and over who have recognized comorbidities.^49^ Few such patients will present for anesthesia. However, drug indications change, and the drug may find use for other diseases.

The current anesthesia restrictions associated with ritonavir deny children of other drugs with considerable value. It is better that the changes in clearance or bioavailability of these drugs are understood so that rational drug choices can be made to tailor drug to the individual patient. Alteration of drug dose, anticipation of duration of effect, timing of administration, use of reversal drugs (e.g., naloxone, flumazenil, sugammadex), and perioperative monitoring would better behoove children undergoing anesthesia.

## 7 REFLECTIVE QUESTIONS

1. How does a drug such as ritonavir, strong inhibitor of CYP 3A, prolong the effects of the antiviral drug, nirmatrelvir?
2. Why should small changes in drug concentration in a drug with a steep concentration‐response relationship have clinically relevant effects?
3. Why should the route of administration have importance for drugs cleared by the liver CYP 3A4 clearance pathway when co‐administered with ritonavir?
4. What pharmacological considerations and monitoring might you consider before starting a general anesthetic a child with COVID‐19 who is taking nirmatrelvir–ritonavir combination therapy? Consider the premedication, induction agent, neuromuscular blocking drug, and opioid.

## FUNDING INFORMATION

This work was funded from institutional resources.

## CONFLICT OF INTEREST

AS and HH have no conflicts of interest to declare. BJA is Associate Editor‐in‐Chief for Pediatric Anesthesia.

## ETHICAL APPROVAL STATEMENT

Human ethics committee approval not required.

## Data Availability

Data sharing not applicable to this article as no datasets were generated or analysed during the current study.
